# Methods to systematically review and meta-analyse observational studies: a systematic scoping review of recommendations

**DOI:** 10.1186/s12874-018-0495-9

**Published:** 2018-05-21

**Authors:** Monika Mueller, Maddalena D’Addario, Matthias Egger, Myriam Cevallos, Olaf Dekkers, Catrina Mugglin, Pippa Scott

**Affiliations:** 1 Institute of Social and Preventive Medicine, University of Bern, Bern, Switzerland; 2 Translational Research Center, University Hospital of Psychiatry, University of Bern, Bern, Switzerland; 3 CTU Bern, Clinical Trials Unit Bern, Bern University Hospital and University of Bern, Bern, Switzerland; 4 Department of Clinical Epidemiology, Leiden University Medical Centre, Leiden, The Netherlands; 5 Department of Clinical Epidemiology, Aarhus University Hospital, Aarhus, Denmark; 6 Department of Pathology and Biomedical Science, University of Otago, Christchurch, New Zealand

**Keywords:** Recommendation, Observational studies, Systematic review, Meta-analysis, Methods

## Abstract

**Background:**

Systematic reviews and meta-analyses of observational studies are frequently performed, but no widely accepted guidance is available at present. We performed a systematic scoping review of published methodological recommendations on how to systematically review and meta-analyse observational studies.

**Methods:**

We searched online databases and websites and contacted experts in the field to locate potentially eligible articles. We included articles that provided any type of recommendation on how to conduct systematic reviews and meta-analyses of observational studies. We extracted and summarised recommendations on pre-defined key items: protocol development, research question, search strategy, study eligibility, data extraction, dealing with different study designs, risk of bias assessment, publication bias, heterogeneity, statistical analysis. We summarised recommendations by key item, identifying areas of agreement and disagreement as well as areas where recommendations were missing or scarce.

**Results:**

The searches identified 2461 articles of which 93 were eligible. Many recommendations for reviews and meta-analyses of observational studies were transferred from guidance developed for reviews and meta-analyses of RCTs. Although there was substantial agreement in some methodological areas there was also considerable disagreement on how evidence synthesis of observational studies should be conducted. Conflicting recommendations were seen on topics such as the inclusion of different study designs in systematic reviews and meta-analyses, the use of quality scales to assess the risk of bias, and the choice of model (e.g. fixed vs. random effects) for meta-analysis.

**Conclusion:**

There is a need for sound methodological guidance on how to conduct systematic reviews and meta-analyses of observational studies, which critically considers areas in which there are conflicting recommendations.

**Electronic supplementary material:**

The online version of this article (10.1186/s12874-018-0495-9) contains supplementary material, which is available to authorized users.

## Background

Many research questions cannot be investigated in randomised controlled trials (RCTs) for ethical or methodological reasons [1], and around 80–90% of published clinical research is observational in design [2, 3]. The Framingham Heart Study, National Child Development Study, and the Dunedin Multidisciplinary Health & Development Study are examples of large observational studies that have provided important information about risk factors and prevention of major public health problems [4–6].

Systematic reviews and meta-analyses synthesise evidence from multiple studies and can potentially provide stronger evidence than individual studies alone. Systematic reviews considering observational data are frequently performed and in a survey of 300 systematic reviews, 64% of the reviews included observational studies [7]. Importantly, synthesis of evidence from observational studies differs from the approach used when examining evidence from RCTs. For example, the process of defining the research question and conducting an adequate literature search is likely to be more iterative than in reviews of RCTs, the risk of bias assessment is different, and decisions around combining results require more careful consideration to avoid precise but misleading results from meta-analysis [8, 9].

Researchers wishing to conduct a systematic review of observational studies should be prepared for the challenges they are likely to encounter. However, guidance on how to conduct systematic reviews of observational studies is not as readily available as guidance for reviews of RCTs. Because observational studies differ in many aspects from RCTs, guidance aimed at reviews of RCTs should be applied with caution to observational studies [10, 11]. A previous methodological guideline published 18 years ago focused on how to report meta-analyses of observational studies rather than how to perform such studies [12]. This guideline also mainly transferred knowledge about evidence synthesis of RCTs directly to evidence synthesis of observational studies. The present article aims to review methodological recommendations on how to conduct systematic reviews and meta-analyses of observational data. It also aims to highlight the similarities and important differences between published recommendations in order to guide future research.

## Methods

We performed a systematic scoping review using methodological approaches previously described [13–15], and following a protocol written prior to starting the review (see Additional file 1).

### Eligibility criteria and definitions

We included published articles if they provided recommendations on at least one key methodological item relating to the conduct of systematic reviews and meta-analyses of observational studies (Table 1). The key items were defined a priori and were based on guidelines on reporting systematic reviews or meta-analyses [10–12]. We included non-randomised studies and quasi-experimental studies or pseudo-RCTs since these studies are often used in the evaluation of healthcare and public health intervention when randomisation is not possible [16]. We considered a recommendation to be any methodological statement to inform the reader how to conduct evidence synthesis of observational studies (e.g. ‘*Any pooled estimate calculated must account for the between-study heterogeneity. In practice, this test has low sensitivity for detecting heterogeneity, and it has been suggested that a liberal significance level, such as 0.1, should be used’)* [16]. We did not consider a recommendation to be a general statement of methodological principles without clear suggestions for the reader (e.g. ‘*The mathematical process involved in this step generally involves combining (pooling) the results of different studies into an overall estimate. Compared with the results of individual studies, pooled results can increase statistical power and lead to more precise estimates of treatment effect*’) [16]. We excluded articles published prior to 1994 since we considered the development of systematic review methods to have started then with the first publication of the Cochrane handbook. We also excluded articles that reported the results of reviews of observational studies without giving recommendations on methodological aspects of how to conduct such a review. Articles that focused on reviews of RCTs, cost effectiveness studies or diagnostic studies were also excluded.

**Table 1:** Methodological key items for systematic reviews or meta-analyses of observational studies

Protocol development	A protocol is written in the preliminary stages of a research synthesis to describe the rational of the review and the methods that will be used to minimise the potential for bias in the review process.
Research question	The research question is defined a priori as for any research project. It sets the scope of the review and guides subsequent decisions about the methods to be used to answer the particular research question.
Search strategy	The search strategy refers to the methods employed to conduct a methodologically sound search and might include information as the data sources used and the specific terms applied in distinct databases. The search locates articles relevant to answer the a priori defined research question.
Study eligibility	Study eligibility is assessed according to pre-defined eligibility criteria related to the study itself such as the study design, the study population, as well as the exposure/s and outcome/s of interest but also to aspects such as the language and year of publication. Usually two reviewers assess each study for eligibility to reduce errors and bias. Specifying which features should be covered by eligibility criteria might be more difficult for observational studies than for RCTs as observational studies cover a broader range of research questions and have more variability in design.
Data extraction	Data extraction is performed according to a standardised form that has been finalised during pilot extraction. Usually two reviewers extract data for each study for eligibility to reduce errors and bias. Data extraction for observational studies might be less straight forward than for RCTs because multiple analyses may have been conducted (e.g. unadjusted and adjusted, with analyses adjusting for different sets of potential confounders), and each observational study design will have different data to be extracted.
Considering different study designs	Before starting evidence synthesis of observational studies, reviewers must consider which study designs to include as well as how to approach the analysis of data from different study designs. This adds complexity over evidence synthesis that considers RCTs only.
Risk of bias assessment	A risk of bias assessment of all primary studies included is important for all systematic reviews and meta-analyses. This assessment allows a better understanding of how bias may have affect results of studies, and subsequently the results of evidence synthesis. Risk of bias assessment of observational studies may be more complex than in RCTs since observational studies are likely to be prone to bias and confounders.
Publication bias	Publication bias needs to be considered in any systematic review and meta-analysis as only about half of all completed research projects reach publication in an indexed journal.
Heterogeneity	The term heterogeneity refers to differences in results between studies. When heterogeneity exists between studies, it is important to understand why as this will alter the conclusions drawn by the review. An exploration of heterogeneity might be particularly important when reviewing observational studies given the range of study designs and the potential risk of bias in observational studies.
Statistical analysis	Statistical analysis in the context of meta-analysis refers to the mathematical analysis and combination of the results of the included primary studies. Important aspects to consider are whether to pool data to provide a single effect in light of observed heterogeneity and how to choose the statistical model to be employed (e.g. fixed or random-effects model). These decisions might need more careful consideration when reviewing observational studies given the range of study designs and the potential risk of bias in observational studies.

### Literature search

We based our literature search on the principle of theoretical saturation [17, 18], with the aim of identifying all relevant recommendations, rather than all relevant articles. We identified the articles by searching electronic databases (Medline and the Cochrane Methodology Register (CMR)) and specific websites of review centres (the Cochrane Library, the Centre for Reviews and Dissemination (CRD), the Campbell Collaboration, the Scottish Intercollegiate Guidelines Network (SIGN), the Agency for Healthcare Research and Quality (AHRQ), the EQUATOR Network, the National Institute for Health and Care Excellence (NICE), the Effective Public Health Practice Project (EPHPP)) and the Joanna Briggs Institute [19–28]. We screened all online issues of specific journals focusing on evidence synthesis (Research Synthesis Methods, Systematic Reviews and Epidemiologic Reviews). To complete our search, we contacted experts in the field for additional references, and added relevant articles referenced in included full texts to the list of potentially eligible papers. We conducted sensitivity searches to define the final search strategy in Medline (Additional file 2). For other databases or websites, we used a combination of the terms “systematic review”, “meta-analysis”, and “observational”. We applied no language restrictions in searches. The initial search was performed in January 2014. Searches were repeated in February 2017, with the exception of the CMR because the database has not been updated since mid-2012.

### Article selection and data extraction

Each title and abstract was screened independently by two reviewers for recommendations on at least one of the key items. The full-texts of all articles considered potentially eligible were then assessed for eligibility. Disagreements regarding eligibility were resolved by discussion with a senior methodologist (M.E., O.M.D. or P.S.).

We randomly assigned all included articles to three pairs of reviewers who independently recorded the key items addressed in the paper and extracted relevant text. Consensus on extracted text was reached by discussion within the pair and disagreements were resolved by consulting a senior methodologist (M.E., O.M.D. or P.S.). We additionally extracted a limited set of standard variables from each included article to summarise the source of recommendations, including the database from which the article was retrieved, the affiliations of the first and last authors (classified as international systematic-review-methods organisation; statistical or epidemiological department; or specialist clinical/health-related department ) and the type of journal (general international medical journal; specialist international medical journal; national medical journal; statistical/epidemiological journal; or systematic review methods journal). We also identified the study design or designs at which each article is aimed [13–15]. We allocated each extracted recommendation to one methodological key item. We did not appraise the methodological quality of the included articles and recommendations because widely accepted standards of systematic reviews and meta-analysis of observational studies are lacking at present. We summarised the data using a descriptive approach and performed qualitative thematic analysis of the recommendations extracted as text.

## Results

### Identification of eligible articles

**Fig. 1:**
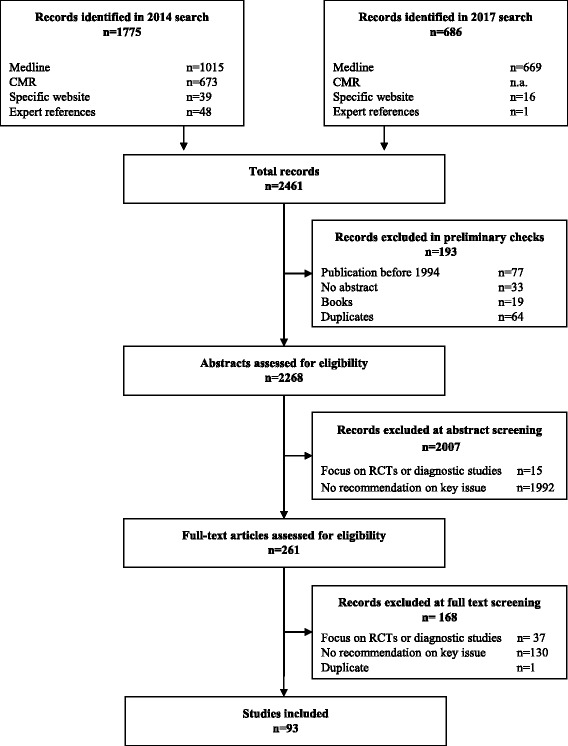
Flow chart of article selection

The searches identified 2461 articles. Electronic databases and websites provided 2412 articles (Fig. 1), and consultation with experts and references from screened full texts added a further 49. After removing 193 items (duplicates, outside publication dates, books), 2268 were screened for eligibility. The most common reason for exclusion was not providing a recommendation on a key item (2122 articles). We included 93 articles.

### Overview of recommendations

Table 2 shows the key items addressed by the recommendations in each article. Only one (1%) of the 93 included articles addressed all key items [29], 56 (60%) articles gave recommendations on two or more key items, and 37 (40%) articles gave specific recommendations on only one key item. Table 3 shows the main topics of recommendations within each key item. See Additional file 3: Table S1 shows the type of journal and author affiliation for each article providing information about the origin of the recommendations. The majority of articles (62%) were published in statistical, epidemiological or systematic review methodological journals followed by 29% in medical journals and 9% in health sciences journals. Of the included articles, 72% were written by authors affiliated with either a systematic review organisation or a statistical/epidemiological department of a university. We found conflicting recommendations for the key items “research question”, “study eligibility”, “considering different study designs”, “risk of bias assessment”, “publication bias” and “statistical analysis” (Table 4).

**Table 2:** Study characteristics and recommendations by key item

Authors, year	Study designs targeted^a^	Protocol	Research Question	Search	Eligibility	Extraction	Study Designs	Risk of Bias	Publication Bias	Heterogeneity	Statistics
Abrams, 1995 [102]	Cohort and case-control	✗	✗	✗	✗	✗	✗	✗	✗	**✓**	✗
Armstrong, 2007 [39]	Observational	**✓**	**✓**	**✓**	**✓**	✗	**✓**	✗	✗	**✓**	✗
Ashford, 2009 [36]	Observational	**✓**	**✓**	**✓**	✗	✗	✗	✗	**✓**	**✓**	**✓**
Austin, 1997 [76]	Cohort and case-control	✗	✗	✗	✗	✗	**✓**	✗	✗	✗	✗
Balshem, 2011 [84]	Observational	✗	✗	✗	✗	✗	✗	**✓**	✗	✗	✗
Blair, 1995 [34]	Observational	**✓**	✗	**✓**	**✓**	**✓**	**✓**	**✓**	**✓**	**✓**	**✓**
Brockwell, 2001 [116]	Not specified	✗	✗	✗	✗	✗	✗	✗	✗	✗	**✓**
Chaiyakunapruk, 2014 [54]	Cohort and case-control	✗	**✓**	**✓**	**✓**	✗	✗	**✓**	✗	**✓**	**✓**
Chambers, 2009 [32]	Case series	**✓**	✗	✗	✗	✗	✗	**✓**	✗	✗	✗
Colditz, 1995 [77]	Cohort and case-control	✗	✗	✗	✗	✗	**✓**	**✓**	✗	**✓**	**✓**
Davey Smith, 1997 [98]	Observational	✗	✗	✗	✗	✗	✗	✗	**✓**	**✓**	✗
Davey Smith, 1998 [96]	Observational	✗	✗	✗	✗	✗	✗	✗	**✓**	✗	**✓**
Doria, 2005 [103]	Observational and RCT	✗	✗	✗	✗	✗	✗	✗	✗	**✓**	**✓**
Dwyer, 2001 [101]	Cohort and case-control	✗	✗	✗	✗	✗	✗	✗	✗	**✓**	**✓**
Egger, 1997a [29]	Observational	**✓**	**✓**	**✓**	**✓**	**✓**	**✓**	**✓**	**✓**	**✓**	**✓**
Egger, 1997b [97]	Observational	✗	✗	✗	✗	✗	✗	✗	**✓**	✗	✗
Fraser, 2006 [58]	Observational	✗	✗	**✓**	✗	✗	✗	✗	✗	✗	✗
Friedenreich, 1994 [31]	Case-control	**✓**	✗	✗	✗	✗	✗	✗	✗	✗	**✓**
Furlan, 2006 [59]	Observational	✗	✗	**✓**	✗	✗	✗	✗	✗	✗	✗
Golder, 2008 [60]	Observational	✗	✗	**✓**	✗	✗	✗	✗	✗	✗	✗
Greenland, 1994 [85]	Cohort and case-control	✗	✗	✗	✗	✗	✗	**✓**	**✓**	**✓**	**✓**
Guyatt, 2011a [95]	Observational	✗	✗	✗	✗	✗	✗	✗	**✓**	✗	✗
Guyatt, 2011b [45]	Observational	✗	**✓**	✗	✗	✗	✗	✗	✗	✗	✗
Guyatt, 2011c [93]	Observational	✗	✗	✗	✗	✗	✗	**✓**	✗	✗	✗
Guyatt, 2011d [106]	Observational	✗	✗	✗	✗	✗	✗	✗	✗	**✓**	✗
Hartemink, 2006 [109]	Observational	✗	✗	✗	✗	✗	✗	✗	✗	✗	**✓**
Haynes, 2005 [57]	Cohort	✗	✗	**✓**	✗	✗	✗	✗	✗	✗	✗
Herbison, 2006 [92]	Observational	✗	✗	✗	✗	✗	✗	**✓**	✗	✗	✗
Hernandez, 2016 [107]	Cohort, case-control and cross- sectional	✗	✗	✗	✗	✗	✗	✗	✗	**✓**	**✓**
Higgins, 2013 [65]	Observational	✗	✗	**✓**	**✓**	✗	**✓**	**✓**	✗	✗	✗
Horton, 2010 [74]	Cross-sectional	✗	✗	✗	✗	**✓**	✗	✗	✗	✗	✗
Ioannidis, 2011 [88]	Observational	✗	✗	✗	✗	✗	✗	**✓**	✗	✗	✗
Khoshdel, 2006 [30]	Observational and RCT	**✓**	**✓**	**✓**	**✓**	✗	✗	**✓**	✗	**✓**	**✓**
Kuper, 2006 [62]	Observational	✗	✗	**✓**	✗	✗	✗	✗	✗	✗	✗
Lau, 1997 [16]	Observational	✗	✗	✗	✗	✗	✗	**✓**	**✓**	**✓**	**✓**
Lemeshow, 2005 [63]	Observational	✗	✗	**✓**	✗	✗	✗	✗	✗	✗	✗
Loke, 2011 [64]	Observational and RCT	✗	✗	**✓**	✗	✗	✗	**✓**	✗	✗	✗
Loke, 2007 [35]	Cohort, case-control and cross- sectional	**✓**	**✓**	**✓**	✗	**✓**	**✓**	**✓**	✗	✗	**✓**
MacDonald-Jankowski, 2001 [46]	Observational and RCT	✗	**✓**	**✓**	**✓**	✗	✗	✗	**✓**	✗	✗
Mahid, 2006 [55]	Observational and RCT	✗	✗	**✓**	**✓**	✗	✗	✗	**✓**	**✓**	**✓**
Manchikanti, 2009 [47]	Observational	✗	**✓**	**✓**	**✓**	**✓**	✗	**✓**	✗	**✓**	**✓**
Martin, 2000 [79]	Cohort and case-control	✗	✗	✗	✗	✗	**✓**	✗	✗	✗	**✓**
McCarron, 2010 [114]	Observational and RCT	✗	✗	✗	✗	✗	✗	✗	✗	✗	**✓**
Moola, 2015 [41]	Observational and RCT	**✓**	**✓**	✗	**✓**	✗	✗	✗	✗	**✓**	**✓**
Moreno, 1996 [80]	Case-control	✗	✗	✗	✗	✗	**✓**	✗	✗	✗	**✓**
Munn, 2015 [72]	Observational	✗	✗	✗	**✓**	✗	✗	✗	✗	**✓**	**✓**
Naumann, 2007 [67]	Not specified	✗	✗	**✓**	✗	✗	✗	✗	✗	✗	✗
Normand, 1999 [48]	Observational and RCT	✗	**✓**	**✓**	**✓**	**✓**	✗	**✓**	**✓**	✗	**✓**
Norris, 2013 [71]	Observational and RCT	✗	✗	✗	**✓**	✗	✗	**✓**	✗	✗	✗
O’Connor, 2014 [42]	Observational	**✓**	**✓**	✗	**✓**	✗	**✓**	✗	✗	**✓**	**✓**
Pladevall-Vila, 1996 [100]	Observational	✗	✗	✗	✗	✗	✗	✗	✗	**✓**	**✓**
Prevost, 2000 [117]	Observational	✗	✗	✗	✗	✗	✗	✗	✗	✗	**✓**
Price, 2004 [49]	Observational	✗	**✓**	**✓**	✗	✗	**✓**	**✓**	**✓**	**✓**	✗
Raman, 2012 [50]	Observational and RCT	✗	**✓**	**✓**	✗	✗	✗	**✓**	**✓**	**✓**	✗
Ravani, 2015 [43]	Observational	**✓**	**✓**	**✓**	**✓**	**✓**	✗	**✓**	✗	**✓**	**✓**
Robertson, 2014 [94]	Observational and RCT	✗	✗	✗	✗	✗	✗	**✓**	✗	✗	✗
Rosenthal, 2001 [51]	Observational and RCT	✗	**✓**	**✓**	✗	✗	**✓**	✗	✗	**✓**	✗
Sagoo, 2009 [33]	Observational	**✓**	✗	**✓**	**✓**	**✓**	✗	**✓**	**✓**	**✓**	**✓**
Salanti, 2005 [87]	Observational	✗	✗	✗	✗	✗	✗	**✓**	✗	**✓**	**✓**
Salanti, 2009 [110]	Observational	✗	✗	✗	✗	✗	✗	✗	✗	✗	**✓**
Sanderson, 2007 [90]	Observational	✗	✗	✗	✗	✗	✗	**✓**	✗	✗	✗
Schünemann, 2013 [40]	Observational	**✓**	**✓**	✗	✗	✗	✗	✗	✗	✗	✗
Shamliyan, 2012 [89]	Observational and RCT	✗	✗	✗	✗	✗	✗	**✓**	✗	✗	✗
Shuster, 2007 [118]	Observational	✗	✗	✗	✗	✗	✗	✗	✗	✗	**✓**
Simunovic, 2009 [38]	Observational and case-control	**✓**	**✓**	**✓**	**✓**	**✓**	**✓**	**✓**	✗	**✓**	**✓**
Smith, 1995 [111]	Observational	✗	✗	✗	✗	✗	✗	✗	✗	✗	**✓**
Souverein, 2012 [81]	Observational and RCT	✗	✗	✗	✗	✗	**✓**	✗	✗	✗	**✓**
Stansfield, 2016 [68]	Not specified	✗	✗	**✓**	✗	✗	✗	✗	✗	✗	✗
Sterne, 2016 [82]	Observational	✗	✗	✗	✗	✗	**✓**	**✓**	✗	**✓**	**✓**
Stroup, 2000 [12]	Observational	✗	✗	**✓**	**✓**	✗	✗	**✓**	✗	**✓**	**✓**
Sutton, 2002a [78]	Observational and RCT	✗	✗	✗	✗	✗	**✓**	✗	**✓**	✗	✗
Sutton, 2002b [99]	Observational and RCT	✗	✗	✗	✗	✗	✗	✗	**✓**	✗	✗
Tak, 2010 [52]	Cohort and case-control	✗	**✓**	✗	✗	✗	✗	**✓**	✗	**✓**	**✓**
Takkouche, 1999 [104]	Observational	✗	✗	✗	✗	✗	✗	✗	✗	**✓**	**✓**
Thomas, 2004 [53]	Observational	✗	**✓**	**✓**	✗	✗	✗	**✓**	✗	✗	✗
Thompson, 2002 [115]	Observational	✗	✗	✗	✗	✗	✗	✗	✗	✗	**✓**
Thompson, 2011 [112]	Observational	✗	✗	✗	✗	✗	✗	✗	✗	✗	**✓**
Thompson, 2014 [69]	Observational	✗	✗	**✓**	✗	✗	✗	✗	✗	✗	✗
Thornton, 2000 [61]	Observational	✗	✗	**✓**	**✓**	✗	**✓**	✗	**✓**	**✓**	**✓**
Tufanaru, 2015 [44]	Observational	**✓**	✗	✗	✗	✗	**✓**	✗	✗	**✓**	**✓**
Tweedie, 1995 [113]	Cohort and case-control	✗	✗	✗	✗	✗	✗	✗	✗	✗	**✓**
Valentine, 2013 [75]	Observational and RCT	✗	✗	✗	✗	✗	**✓**	**✓**	✗	✗	✗
Verde, 2015 [83]	Observational and RCT	✗	✗	✗	✗	✗	**✓**	✗	✗	✗	**✓**
Weeks, 2007 [108]	Cohort and case-control	✗	✗	✗	✗	✗	✗	✗	✗	✗	**✓**
Wells, 2013 [37]	Observational and RCT	**✓**	✗	✗	**✓**	✗	**✓**	**✓**	✗	✗	✗
West, 2002 [91]	Cohort and case-control	✗	✗	✗	✗	✗	✗	**✓**	✗	✗	✗
Wille-Jorgensen, 2008 [56]	Observational and RCT	✗	✗	**✓**	✗	✗	**✓**	**✓**	**✓**	**✓**	**✓**
Winegardner, 2007 [66]	Observational, Cohort and case-control	✗	✗	**✓**	✗	✗	✗	**✓**	✗	**✓**	**✓**
Wong, 2008 [86]	Observational	✗	✗	✗	✗	✗	✗	**✓**	✗	✗	✗
Wong, 1996 [70]	Cohort	✗	✗	✗	**✓**	✗	**✓**	**✓**	**✓**	**✓**	**✓**
Zeegers, 2000 [105]	Observational	✗	✗	✗	✗	✗	✗	✗	✗	**✓**	**✓**
Zingg, 2016 [73]	Observational and cohort	✗	✗	✗	**✓**	✗	**✓**	**✓**	✗	✗	**✓**
Zwahlen, 2008 [8]	Observational	✗	✗	✗	✗	✗	**✓**	✗	✗	**✓**	**✓**

^a^Describes the study designs toward which articles target their recommendations. Articles that target “observational” or “non-randomised” studies are categorised under observational. “Not specified” refers to articles that do not name study designs, but provide recommendations applicable to observational studies

**Table 3:** Summary of recommendations from 93 publication by key item

Key item	No of articles providing recommendation	Topic of recommendation	N articles addressing area (%)^a^
Protocol development	16	Need for protocol to be written in advance	12 (75%)
		Items to be included in protocol	11 (69%)
Research question	20	Scope of research question	20 (100%)
Search strategy	33	General methods for conducting searches in context of observational studies	22 (67%)
		Specific challenges in searching for observational studies	12 (36%)
Study eligibility	22	Specifying eligibility criteria	22 (100%)
		Assessment of eligibility	6 (27%)
Data extraction	9	Methods for data extraction	9 (100%)
Dealing with different study designs	25	Inclusion of different study designs in a single review	10 (40%)
		Combining results from different study designs in a single meta-analysis	15 (60%)
Risk of bias assessment	39	Methods to assess the risk of bias in individual studies	39 (100%)
Publication bias	20	Inclusion of unpublished studies	5 (25%)
		Methods to assess publication bias	7 (35%)
Heterogeneity	39	Measurement of heterogeneity	39 (100%)
		Exploring potential causes of heterogeneity	16 (41%)
Statistical analysis	52	Deciding to combine results in a single effect estimate	20 (38%)
		Choosing fixed or random effects meta-analysis	16 (31%)

^a^Percentages do not add up to 100% because articles can contribute recommendations to more than one topic and only the most frequent areas of recommendation for each key item are listed

**Table 4:** Key item with conflicting recommendations

	Recommendations in favour	Recommendations against
Research question		
Should we formulate the research question as precise as possible?	“A focused research question is essential. The question that is asked needs to be as scientifically precise as possible.” [51] “While others (e.g., EPPI-Centre) have opted to answer very broad questions in their reviews, we have chosen to keep our questions quite specific. We have done this for two reasons. First, practitioners and policymakers want answers to specific questions, and so our reviews and their summary statements provide this. Second, keeping questions specific limits any one literature search and retrieval. Given that the “hit” rate for relevant articles in an electronic search regarding public health topics is about 10%, any review requires a lot of reviewer time to select the relevant articles from those identified. When topics are broad, the “hit” rate can be even lower, requiring more resources.” [53]	“Thus, questions that the review addresses may be broad or narrow in scope, with each one of them associated with their own advantages and disadvantages. While the questions may be refined based on the data which is available during the review, it is essential to guard against bias and modifying questions, as post-hoc questions are more susceptible to the bias than those asked a priori and data-driven questions can generate false conclusions based on spurious results.” [47] “A review needs to focus on meaningful and not trivial outcomes. The chosen focus of a review, whether broad or narrow, will not, in itself affect the quality of the review but, it will impact on its relevance.” [49] “The research question about safety and tolerability in a review may be broad or narrow in scope. […] In general, reviewers who have already identified important safety concerns (for instance, from the knowledge of the pharmacology, or anatomical site of the intervention) should carry out a narrow-focused evaluation covering particular aspects of the relevant adverse effects. On the other hand, reviewers who are not aware of any specific safety problems, could start with a general overview of the range of adverse effects associated with an intervention. A widely scoped review may be part of an initial evaluation which eventually throws up specific safety issues that merit further focused study.” [35]
Study eligibility		
Should we include studies of all languages?	“Ideally, it would be best to include all studies regardless of language of publication. However, for practical reasons, many meta-analyses limit themselves to English language studies. Although this decreases the number of studies, it does not appear to bias the effect size”. [30]	“Including papers in all languages may actually introduce more bias into a meta-analysis”. [61]
Should we avoid multiple inclusions?	“authors must be careful to avoid the multiple inclusion of studies from which more than one publication has arisen”. [61]	“It is important that each entry in a meta-analysis represents an independent sample of data. Thus, for example, multiple reports of the same study need to be merged to obtain a single “best” answer for that study” [33]
Considering different study designs		
Should we include both RCT and NRS in a single systematic review?	“When both randomized and non-randomized evidence are available, we favor a strategy of including NRS and RCTs in the same systematic review but synthesizing their results separately.” [75] “When an adverse event is rare or occurs a long time after intervening, including NRS in systematic reviews may be desirable because randomized controlled trials (RCTs) often have inadequate power to detect a difference in harm between intervention and control groups and commonly do not follow up participants in the long term …Another reason to include NRS in a systematic review is that there might be no or very few RCTs, and there may be a need to synthesize the best available evidence.” [75] “Systematic reviews that evaluate vaccine safety will need to expand to include study designs beyond RCTs. Randomisation is the only way to control for all unknown confounders, thereby minimising the effects of bias on the results. Only limited empirical evidence is available on the impact that non-randomised study designs may have on the measurement of adverse events.” [49] “Under ideal circumstances, studies of different designs should be included.” [34]	“Ideally, researchers should consider including only controlled trials with proper randomisation of patients that report on all initially included patients according to the intention to treat principle and with an objective, preferably blinded, outcome assessment.” [29] “Where RCTs (including cluster RCTs) are available to answer questions of effectiveness or efficacy they should be included in your review. This type of study design has the greatest potential for maximising internal validity. RCTs may not be available, and in these circumstances, non-RCTs are likely to represent the best available evidence and should be included” [39].
Should we pool results of different study designs in a single meta-analysis if results are similar over the different study designs?	“If the meta-analysis includes some randomized experiments and some observational studies, we can meta-analyze them separately and combine their results if they are quite similar, borrowing strength for the randomized experiments from the similar results of the nonrandomized studies.” [51] “The contribution of study design to heterogeneity in the effect estimates should be analysed and separate meta-analysis should be conducted by study design when the effect estimates systematically vary by design.” [34] “From these examples, we conclude that an initial stratification of results by study design is useful. A combined analysis should adjust for design features if there is heterogeneity across study designs or, alternatively, results should be reported separately for each design, and further exploration may be warranted to understand the sources of the differences.” [77]	“Generally, separate meta-analyses should be performed on studies of different designs. It is not usually advisable to combine studies of different designs in a single meta-analysis unless it can be determined that study design has little or no influence on study characteristics such as quality of data, specificity of exposure, and uniformity of diagnoses. In reality, study design is usually one of the most important determinants of data quality, exposure specificity, and diagnostic criteria. Similarly, studies with very different statistical techniques, different comparison populations, or different diagnostic categories should generally not be lumped into a single analysis.” [70] “Therefore, in most situations we do not recommend combining cohort and case-control studies in a single meta-analysis. The meta-analysis should at least be stratified by study design.” [70] “We favor a strategy of including NRS and RCTs in the same systematic review, but synthesizing their results separately. Including NRS will often make the limitations of the evidence derived from RCTs more apparent, thereby guiding inferences about generalizability, and may help with the design of the next generation of RCTs.” [75] “While there is absence of overall consensus on the reporting of nonrandomized studies, there is general agreement that combining data between nonrandomized and randomized studies is methodologically flawed, and that multilevel extrapolations should be avoided.” [56]
Risk of bias assessment		
Should we use scales and summary scores to assess the quality of studies?	“The methodological quality of the recruited studies must be checked before analysis. There are several checklists and score systems to facilitate decision about the quality of a study”. [30] “The idea of computing some sort of quality score is attractive” [77]. “… a chosen quality scoring system, especially if oriented to measuring biases, might be used to adjust results” [77]	“We do not recommend the use of quality scoring for the simple reason that it would be impossible to treat different study characteristics … that are related to quality as if they are of equal importance or interchangeable and can be measured by a single score”. [70] “Most methodologists hate this. There is tremendous variability in calculating aggregate quality scores. Two biases may cancel out, have independent effects or multiplicative impact on the results”. [88] “Our broad recommendations are that tools should (i) include a small number of key domains; (ii) be as specific as possible (with due consideration of the particular study design and topic area); (iii) be a simple checklist rather than a scale and (iv) show evidence of careful development, and of their validity and reliability”. [89] “Finally, I wholeheartedly condemn quality scores because they conflate objective study properties (such as study design) with subjective and often arbitrary quality weighting schemes. Use of such scores can seriously obscure heterogeneity sources and should be replaced by stratification or regression analyses of the relation of study results to the items or components of the score”. [85] “It adds to the previous evidence that contemporary quality scores have little or no value in improving the utility of a meta-analysis. Indeed, they may introduce bias, because you get a different answer depending on which quality score you use. In addition, none of the quality scores considered clearly performed better than others when using large trials as a reference standard”. [92]
Publication bias		
Should we assess publication bias with a funnel plot?	“Bias can be detected visually by drawing a funnel plot”. [55] “Publication bias is difficult to eliminate, but some statistical procedures may be helpful in detecting its presence. An inverted funnel plot is sometimes used to visually explore the possibility that publication bias is present”. [16] “A graphic device known as funnel plot can be employed to detect the presence of publication bias”. [48] “The likely presence or absence of bias should be routinely examined in sensitivity analysis and funnel plot”. [97]	“Important, but graphical attempts to detect publication bias can be influenced by the subjective expectations of the analyst”. [85]
Statistical analysis		
Should we use statistical measures of heterogeneity to decide on statistical model?	“Failing to reject the null-hypothesis assumes that there is homogeneity across the studies and differences between studies are due to random error. In this case a fixed-effect analysis is appropriate” [55]. “… when statistical heterogeneity is present in a meta-analysis, a random effects model should be used to calculate the overall effect” [66].	“In taking account of heterogeneity when summarizing effect measures from observational studies many authors recommend formal tests of heterogeneity. However, the available tests often lack statistical power. This means that the possible existence should be considered even where the available tests fail to demonstrate it” [101]. “… the decision as to whether estimated differences are large enough to preclude combination or averaging across studies should depend on the scientific context, not just statistical significance” [34].

### Protocol development

Sixteen articles (17%) provided recommendations on the key item “protocol development” (Table 3), [29–44] with publication dates between 1994 and 2015 (median year of publication 2009). The majority of articles emphasised the importance of developing a protocol for systematic reviews. They gave similar recommendations, but differed slightly on the reasons for writing a protocol and on the elements to address in the protocol. The most common reason given for writing a protocol was to reduce bias in the selection of the studies by pre-specifying the study selection criteria [37, 38, 40, 42]. Further reasons mentioned were to ensure replicability [34], and to document all procedures used in the review [31]. The articles recommended that the protocol should state the objectives, hypotheses to be tested and the rationale of the review, [29] and that it should describe eligibility criteria [29, 33, 35, 36, 38, 39, 41], define the type of studies to be included [35, 37, 42, 44], and give the reason when including observational studies in the review [35, 37, 40]. Additionally, it was recommended that the protocol should define the methods to be used for risk of bias assessment, meta-analysis and exploration of heterogeneity [41, 42, 44].

### Research question

Twenty articles (22%) gave recommendations on the key item “research question” [29, 30, 35, 36, 38–43, 45–54], with publication dates between 1997 and 2015 (median year of publication 2009). All articles described the research question as the essential basis that defines the scope and justifies the rationale of a systematic review. Aspects that were frequently mentioned as important to address were the population, exposures or interventions, and outcomes [38–41, 43, 47, 48, 50, 54]. Two articles recommended that the review question state which study designs will be considered in the review [47, 48]. There was some disagreement (Table 4) whether the research question should be specific (narrowly formulated) [51, 53], or general (broadly formulated) [35, 47, 49]. One article stated that *“A focused research question is essential. The question that is asked needs to be as scientifically precise as possible”* [51] while another countered that *“A review needs to focus on meaningful and not trivial outcomes. The chosen focus of a review, whether broad or narrow, will not, in itself affect the quality of the review but, it will impact on its relevance”* [49].

### Search strategy

Thirty-three articles (35%) made recommendations about the key item “search strategy” [12, 29, 30, 33–36, 38, 39, 43, 46–51, 53–69], with publication dates between 1995 and 2016 (median year of publication 2007). The majority of articles discussed aspects general to systematic reviews including advantages and limitations of different literature sources and databases, search tools, the importance of identifying unpublished studies, literature searching techniques including how to build a search string and reporting [12, 29, 33–36, 46, 48, 50, 51, 53, 54, 57, 59–62, 67, 68].

About one third of the articles acknowledged that searching for observational studies requires additional time and resources because of lack of specific search filters and poorly established or inconsistently used indexing terms [38, 39, 47, 49, 56, 58, 59, 64, 65]. Finding all available information may not be as important in reviews of observational studies as in reviews of RCTs [43]. One article stated that “*Reporting of studies in the titles and abstracts infrequently used explicit terms that describe study design. Terms such as case series, cohort, observational, non-random and non-comparative (including variations of these terms) appeared in only a small proportion of records and hence had low sensitivity*” [58]. Because of this and insufficient indexing of observational studies, often a large number of studies are retrieved in searches leading to an inefficient use of resources.

Although there were no direct conflicts between recommendations given in different articles, numerous strategies were presented for searching effectively. For example, one recommendation was to build the search strategy using terms specific to the intervention (e.g. drug name, generic or trade name) and for the study designs when conducting reviews of adverse effects [64]. Another recommendation was to create two different search terms: one for older dates with no limits on study design and the other for more recent dates, after study design search terms were introduced in databases, with study design restrictions applied [59]. One additional article explored selecting search terms using semantic concept recognition software to supplement search term selection by experts [69].

### Study eligibility

Twenty-two articles (24%) provided recommendations on the key item “study eligibility”, [12, 29, 30, 33, 34, 37–39, 41–43, 46–48, 54, 55, 61, 65, 70–73] with publication dates between 1995 and 2016 (median year of publication 2009).

Many recommended that the eligibility criteria need to be pre-specified [30, 33, 38, 39, 47, 55, 65] and that the rationale for defining the eligibility criteria should be explicitly justified [38, 39, 65], unambiguous [65], and derived from the review question [47]. Similar to reviews of RCTs, it was suggested that criteria should be defined in terms of population, interventions, outcomes and study design of interest [47, 54], but a modified version for reviews of observational studies was also proposed: condition, context and population (“CoCoPop”) [72]. One article highlighted that providing a rationale for the eligibility criteria and *“showing how those criteria may minimize potential biases and confounding”* is crucial [38]. Another article recommended that inclusion criteria, particularly with regard to eligible study designs, may differ for different outcomes examined in the same review [42]. Five articles gave recommendations about how to assess eligibility: it should be blind [29, 30, 46], independent [29, 33, 46, 48], and performed by two reviewers [29, 30, 33, 48]. One article recommended using a panel of experts to decide on the inclusion status of a study [48].

We found contradictory recommendations on language of publication, width of eligibility criteria, assessment of full text or abstract to establish eligibility and on how to proceed with duplicates (Table 4). One article recommended including *“all studies regardless of language of publication”* [30], whereas another suggested *“including papers in all languages may actually introduce more bias into a meta-analysis”* [61]. Regarding the width of eligibility criteria, some authors suggested that broad criteria could maintain generalisability [12, 38, 54], while others advocated that narrow criteria might reduce between study heterogeneity [46, 54]. One article recommended basing the decision on abstracts [55], while another article stated that abstracts carry not enough information to determine eligibility and consultation of full-texts is necessary [65]. Some authors suggested that *“authors must be careful to avoid the multiple inclusion of studies from which more than one publication has arisen”* [61], while others recommended merging multiple reports of the same study to obtain a single “best” answer or including the most recent and most complete study [33, 70].

### Data extraction

Nine articles (10%) made recommendations on how to perform data extraction [29, 33–35, 38, 43, 47, 48, 74], with publication dates between 1995 and 2016 (median year of publication 2009). It was generally accepted that data extraction should be performed using a standardised form [29] to avoid omissions [74]. Several articles provided information on the type of information to be extracted [29, 38, 43, 47, 48] such as characteristics of the population and the study setting including environmental and cultural factors; [47, 48] details of the intervention [47], exposure [34], and outcome including sample size, point estimate and standard error; [34, 47, 48] as well as elements of methodological study quality [29, 47]. One article specifically recommended extracting and analysing adjusted results for observational studies, since confounding is expected in any observational study [38]. Several articles recommended that the data should be extracted by two independent reviewers to avoid errors [29, 33], or that blinding those assessing methodological quality to the names of the authors, their institutions and the names of journals would lead to more consistent extraction [29, 34]. Regarding adverse effects it was noted that “*no mention of adverse effects does not necessarily mean that no adverse effects occurred. It is usually safest to assume that they were not ascertained or not recorded: authors must choose whether to exclude the study from the adverse effect analysis or, exceptionally, to include it on the assumption that the incidence was zero*” [35].

### Considering different study designs

Twenty-five (27%) articles gave recommendations on item “considering different study designs” [8, 29, 34, 35, 37–39, 42, 44, 49, 51, 56, 61, 65, 70, 73, 75, 76, 77–83], with publication dates between 1995 and 2016 (median year of publication 2007). Most articles considered the combination of RCTs and non-randomised designs [29, 35, 38, 39, 49, 51, 56, 61, 75, 81]. Only five articles referred to the combination of different non-randomised studies [38, 70, 75, 79, 80]. Recommendations were made mainly on 2 topics: whether different study designs should be included in a systematic review; [29, 34, 37–39, 49, 56, 65, 73, 75] and whether different study designs should be analysed together in a single meta-analysis [8, 34, 35, 37, 38, 42, 49, 51, 56, 61, 70, 75–78]. There was substantial disagreement with regard to these two questions (Table 4).

One article recommended neither including different study designs in systematic reviews nor combining their results in meta-analyses [29]. Five articles discouraged combining different study designs in meta-analyses but saw the added value of including different designs in a systematic review [35, 38, 56, 61, 75]. There was agreement that there was a balance between the availability of data and the introduction of additional biases inherent in observational studies. Articles differed on how the decision should be made. Some offered a broad statements such as *“Any comparative study design such as cohort studies, case-control studies, case-only methods, interrupted time series, historically controlled trials, case-cross-over and epidemiological studies, etc. should all be considered for inclusion if they can help answer the research questions”*, [49] or *“Ideally, researchers should consider including only controlled trials with proper randomisation of patients that report on all initially included patients according to the intention to treat principle and with an objective, preferably blinded, outcome assessment”* [29]. Two recent articles advocated deciding on a case-by-case basis [37, 65]. One article stated that “*Review authors should …. consider carefully, based on their background knowledge of the literature, what kinds of NRS will best allow the systematic review to address the PICO question specified in the review protocol”* [38]. The other article recommended that “*Review authors might need to set different eligibility criteria for different research questions within a review*” [65]. Workload, which is generally higher when observational studies are included, was also mentioned as a factor in the decision of which study designs to include [75].

Authors agreed that combining different study designs in a meta-analysis can lead to misleading results if done without careful consideration [35, 38, 56, 70, 75, 76]. Some authors pointed out that *“a meta-analysis may give a precise estimate of average bias, rather than an estimate of the intervention’s effect”* and that “*heterogeneity between study results may reflect differential biases rather than true differences in an intervention’s effect”* [75]. Some authors agreed that study design should be investigated as a potential source of between study heterogeneity [8, 34, 42, 77], and others recommended presenting meta-analyses for each study type alongside results from an overall meta-analysis [42, 83]. There was, however, little consensus on how to deal with heterogeneity introduced by different study designs. Some authors suggested that results should be combined in a single meta-analysis only in the absence of marked heterogeneity [34, 51]. Two articles recommended stratifying by study design if heterogeneity is large and adjusting for study design if heterogeneity is small [51, 77]. Another article stressed that methods to appropriately combine data from multiple study designs need further development [78].

### Risk of bias assessment

Thirty-nine articles (42%) made recommendations on the assessment of quality and risk of bias of individual studies, [12, 16, 29, 30, 32–35, 37, 38, 43, 47–50, 52–54, 56, 64–66, 70, 71, 73, 75, 77, 82, 84–94] with publication dates between 1994 and 2016 (median year of publication 2009). The term “risk of bias” was specifically used in 18 articles, which were published in more recent years (median year of publication 2012, compared to 2007 in the other articles). Several articles made a distinction between risk of bias of individual studies and the quality of evidence provided by a review. For example, in the GRADE system the quality of a body of evidence may be affected not only by a high risk of bias in individual studies but also by other aspects that affect imprecision of estimates, inconsistency of results from different studies, indirectness of study results (i.e. lack of applicability), and publication bias [84, 93].

All articles recommended that the risk of bias of observational or non-randomised studies included in systematic reviews and meta-analyses should be systematically assessed so that the strength of the body of evidence can be gauged. As one article puts it, *“without appraising each study, it would be impossible to determine to what extent bias and/or confounding had influenced the results”* [50]. The need for complete reporting of all relevant methodological aspects as a prerequisite to assessing the risk of bias of studies was highlighted in 10 articles, [16, 33, 35, 47, 50, 56, 86, 87, 90, 91] and was the focus of recommendations in a publication from the Meta-analysis Of Observational Studies in Epidemiology (MOOSE) group [12]. There was also widespread agreement about the lack of empirical evidence on what study characteristics are most important when assessing the risk of bias of observational studies, and that no widely accepted instrument exists. ROBINS-I, a recently published tool, provides a framework for evaluating the risk of bias in non-randomised studies of interventions [82]. The tool views each study as an attempt to mimic a hypothetical randomised trial, and provides an overall risk of bias judgement across seven bias domains for each outcome.

There was disagreement on how the risk of bias should be assessed (Table 4). Some articles suggested using a quality scale and a summary score calculated for each study while other articles advocated a component approach. The component approach recommends the development of a set of items, guided by the domains of bias most relevant to the eligible studies, and suggests assessing each item separately, without calculating any summary score. The majority of articles advised against the use of scales but 12 articles recommended their use [29, 30, 48–50, 52, 53, 56, 66, 77, 86, 91]. The articles recommending a component approach were published more recently than those recommending the use of scales and summary scores.

### Publication bias

Twenty (22%) articles reported on item “publication bias” [16, 29, 33, 34, 36, 46, 48–50, 55, 56, 61, 70, 78, 85, 95–99], with publication dates between 1994 and 2012 (median year of publication 2001).

A frequently raised issue was whether publication bias could be minimised by the inclusion of unpublished studies. An explicit recommendation to search for and include unpublished studies was given in several articles [34, 48, 50, 61, 96], with the argument that the results of unpublished studies may differ systematically from published studies. One article recommended “*As a first step towards eliminating publication bias, the meta-analyst needs to obtain information from unpublished research”* [48]. However, some authors suggested that the inclusion of unpublished studies could also introduce bias due to poor methodological quality on non-peer reviewed articles: *“the quality of unpublished reports must be closely scrutinized since they presumably have not undergone the same kind of peer review as published literature”* [34].

Since *“journals are typically more likely to publish results that establish a difference than those that do not”* [48], and publication bias is difficult to eliminate, different articles advise to graphically examine its presence using funnel plots, or to assess it with statistical tests [16, 36, 48, 55, 85, 97, 99]. However, some of these articles also raised concerns when using funnel plots or statistical tests. They pointed out that “*detecting bias via funnel plot is not as obvious as it might appear. There may be several types of biasing mechanism present at any given time; for example, there may be both a bias in publishing results from small studies (even significant) as well as against publishing non-significant results…”* [48], and that “*this examination is important but can be influenced by the subjective expectation of the analyst”* [85]. Others highlighted that results might vary with the choice of outcome scale (e.g. odd ratios vs. risk differences) [99].

There was little discussion about whether reviews of observational studies were more prone to publication bias than reviews of RCTs. One article noted that “*in some instances RCTs may be more susceptible to dissemination bias than non-RCTs. For example, for topics with strong financial interest, RCTs are more likely to be funded by sponsors with competing interests, and hence perhaps more likely to publish if favorable results are obtained*” [99].

### Heterogeneity

Thirty-nine articles (42%) provided recommendations on heterogeneity and its assessment, [8, 12, 16, 29, 30, 33, 34, 36, 38, 39, 41–44, 47, 49–52, 54–56, 61, 66, 70, 72, 77, 82, 85, 87, 98, 100–107] with publication dates between 1994 and 2016 (median year of publication 2006). All articles agreed that examination of heterogeneity is a central feature of meta-analysis of observational studies. As one article stated *“a carefully conducted meta-analysis should include assessments of the assumption of homogeneity and, where heterogeneity is found to exist, a careful analysis of its sources should be undertaken*” [34]. Sources of heterogeneity between studies can relate to design (e.g. case-control studies vs control studies, different follow-up periods), to risk of bias (e.g. blinded vs unblinded outcome assessment) or clinical characteristics of included study populations (e.g. different age distributions between studies) [71, 98]. Heterogeneity can be explored using statistical measures such as I^2^- and Tau^2^-or Q-statistics, and in meta-regression analyses [54, 72, 105].

One common recommendation was to explore heterogeneity in sensitivity analyses, comparing studies stratified by design or clinical features [12, 16, 29, 34, 39, 51, 55, 56, 66, 77, 85, 100, 102]. Many recommended to define these subgroups a priori to reduce the risk of false positive findings [33, 52, 77, 106]. One article recommended exploring study heterogeneity by leaving out one or more studies from the analyses and comparing results with the main analysis including all studies [55]. A variation of this recommendation was to exclude studies that are at high risk of bias [42, 44, 54, 73, 82].

### Statistical analysis

Fifty-two articles (56%), provided recommendations on the statistical analysis in the context of meta-analysis of observational studies [8, 12, 16, 29–31, 33–36, 38, 41–44, 47, 48, 52, 54–56, 61, 66, 70, 72, 73, 77, 79–83, 85, 87, 96, 100, 101, 103–105, 107–118], with publication dates between 1994 and 2016 (median year of publication 2006). Two main issues were considered: whether to pool results in a single effect estimate; and the choice between a fixed and a random effects model. Other topics included meta-regression, while few articles focused on more specific statistical issues, such as dose-response analysis [81, 109, 111, 113], credibility ceilings [110], bias adjustment [112], hierarchical models [117], or regression bias [108].

Many considered heterogeneity important for the decision whether to pool the data [34, 36, 47, 55, 56, 66, 101, 103]. However, disagreement exists on how pooling should be done. Three not mutually exclusive approaches can be distinguished. First, a test-based approach: perform a test for heterogeneity, or analogously, use a defined I^2^ threshold, to decide whether data should be combined, and with which model. Authors recommended not to pool if heterogeneity is too high [35, 38, 47, 85, 103], and use statistical measures of heterogeneity to choose between random-effects (in case of heterogeneity) and fixed-effect models [55, 66]. For example: “*Failing to reject the null-hypothesis assumes that there is homogeneity across the studies and differences between studies are due to random error. In this case a fixed-effect analysis is appropriate*” [55]. This approach was, however, often criticised because of low power to detect heterogeneity in case of few (< 10) included studies [52, 77, 101, 104, 116, 118]. Second, the use of a random-effects model for meta-analysis of observational studies by default was frequently recommended [8, 16, 29, 34, 70, 77, 100, 101]. Although a random-effects model takes heterogeneity into account statistically, the drawback is that it may *“divert attention from key sources of heterogeneity, […], such summaries should only be used when important heterogeneity remains after thorough search of the sources of heterogeneity”* [85]. A third approach takes clinical as well as statistical considerations into account when deciding on pooling and the choice of the model [31, 38, 41, 44, 47, 61, 116], because “*statistical tests can not compensate for lack of common sense, clinical acumen and biological plausibility”* [16]. A quote from 20 years ago is thus still pertinent today “*Consensus is needed on how to conduct meta-analyses of observational studies and the methods to be used in the presence of heterogeneity”* [100].

## Discussion

We found ninety-three articles that provided recommendations on how to conduct systematic reviews and meta-analyses of observational studies. Only one article, published in 1997, addressed each of the 10 methodological aspects we examined [29]. Many recommendations for reviews of observational studies were uncritically transferred from reviews of RCTs. Articles giving recommendations for evidence synthesis of observational studies were difficult to locate and frequently gave contradictory recommendations. A comprehensive guidance document on how to conduct evidence synthesis of observational studies is lacking. The most important areas of disagreement particularly relevant to evidence synthesis of observational studies were the width of the research question to be addressed; considering randomised and non-randomised studies in the same review; pooling of results of randomised and non-randomised studies in one meta-analysis; and assessment of quality of observational studies using summary scores. These areas warrant further methodological research.

A strength of the present study is the systematic search and synthesis of existing methodological recommendations on how to conduct systematic reviews and meta-analyses of observational studies. The systematic approach included extensive searches to identify relevant recommendations, eligibility assessment and text extraction by two reviewers, and the structured summary of recommendations according to a priori defined key items or topics [10–12]. Another strength is that we included the key item of whether different study designs should be combined, which is more relevant in evidence synthesis from observational studies than in evidence synthesis from RCTs. Locating methodological papers in electronic databases was challenging: relevant articles may be indexed differently and there is no key word to search for this type of article [18]. To overcome this problem we used broad search terms in Medline and also searched multiple other sources and contact experts in the field. We acknowledge that by including articles based on title and abstract we may have missed some relevant articles. However, our search was guided by the methodological principle of theoretical saturation [17, 18]. Theoretical saturation suggests that identifying every article is not required in methodological research. Once a set of methodological articles covering all relevant methodological aspects is identified, additional searches add little to the ideas already present in the initial set of articles. We excluded books from our review and therefore did not include the Cochrane handbook [10]. The main focus of the Cochrane handbook is the synthesis of evidence from RCTs. We screened the sections on observational studies and were unable to find additional recommendations not covered by the articles included in our review. We did not assess the soundness of recommendations but instead reported an inventory of recommendations, with a focus on contradictory statements indicating where further clarification and research is needed. However, we reported the source of each included article (see Additional file 3: Table S1) to facilitate the critical appraisal of recommendations by the reader [13]. Finally, we refrained from in depth-discussions of statistical methods, which was beyond the scope of the present article.

In light of the increasing use of observational data in systematic reviews and meta-analyses [7], it is crucial for reviewers to have access to sound methodological advice on how to conduct systematic reviews and meta-analyses of observational data. Previous initiatives have primarily focused on improving the reporting of reviews of observational studies [11], or observational studies themselves [12]. Recommendations on reviews of observational studies should be specific to such reviews, rather than being presented as a variation of the “standard” RCT review. The bulk of advice available to reviewers is focused on RCTs, and although many of the procedures are similar in reviews of observational studies, there are important specific considerations such as the choice of eligible study designs, the approach to risk of bias assessment, the special attention to sources of heterogeneity and the choice of statistical methods. It is often unclear whether the results of meta-epidemiological research on systematic reviews of RCTs can be extended to observational studies. Although many authoritative articles providing sound advice exist, such as those included in this review by Greenland [85], Egger et al. [29, 97], and a series in Research Synthesis Methods [37, 40, 65, 71, 75], the inexperienced reviewer may find them difficult to identify among the many other papers with contradictory advice.

Efforts are needed to provide informative, user-friendly and readily available guidance on how to conduct systematic reviews and meta-analyses of observational studies. Emphasis should be placed on producing a single, comprehensive guidance document giving recommendations on the key items outlined in the current review and specifically addressing areas in which we found conflicting recommendations. The guidance provided in the document should be based on a consensus among methodological experts and give authoritative advice in the areas of conflicting recommendations that we identified in this review. The document should ideally include illustrative examples of good practice to guide researchers who are not expert reviewers. Disagreement on methodological advice as identified by this systematic scoping review may also indicate where additional methodological research is needed. Finally, improving the indexing of methodological articles in bibliographic databases and the open access publication of such articles in journals would be helpful.

## Conclusion

We found that many recommendations on how to systematically review and meta-analyse observational studies were taken from advice on reviews of RCTs, neglecting the many methodological differences between these types of study designs. There is considerable disagreement on how systematic reviews and meta-analyses of observational studies should be done, and an urgent need for a comprehensive source of sound methodological guidance that critically considers areas in which we found conflicting recommendations.

## Additional files

Additional file 1: Protocol. (PDF 424 kb)

Additional file 2: Medline search terms. (PDF 100 kb)

Additional file 3: **Table S1.** Sources of recommendations. (PDF 123 kb)

## Abbreviations

AHRQ: The Agency for healthcare research and quality
CMR: Cochrane methodology register
CoCoPop: Condition, context and population
CRD: The centre for reviews and dissemination
EPHPP: The effective public health practice project
MOOSE: Meta-analysis of observational studies in epidemiology
NICE: The National Institute for Health and Care Excellence
NRS: Non-randomised study
PICO: Population, intervention, comparison, outcome
RCT: Randomised controlled trial
SIGN: The Scottish Intercollegiate Guidelines Network
