# Supplementary material for: Methods to systematically review and meta-analyse observational studies: a systematic scoping review of recommendations
Source: BMC Med Res Methodol. 2018 May 21;18:44. doi: 10.1186/s12874-018-0495-9 (PMC5963098; doi:10.1186/s12874-018-0495-9)
Supplement: Supplementary file 1 — Protocol. (PDF 424 kb) [file 12874_2018_495_MOESM1_ESM.pdf]

Guidelines on performing systematic reviews and meta-analyses of observational studies

Systematic review protocol

January 2014

Contents

Introduction 3

Objectives 4

Definition of guideline 4

Methods 5

Search strategy 5

Eligibility 6

Data extraction 6

Summarizing the results 7

References 8

Amendments 9

Introduction

Many research questions, especially those addressing issues such as disease diagnosis or prognosis, adverse effects or harmful risk factors, cannot be investigated in randomized trials due to ethical or methodological reasons. The Framingham Heart Study (http://www.framinghamheartstudy.org/) and the Nurses' Health Study (http://www.channing.harvard.edu/nhs/) are two examples of how long-term observational research can make important contributions to our knowledge of risk factors and prevention of major public health problems. Additionally, when compared to carefully monitored and followed-up randomized controlled trials, observational studies may better reflect what effects occur in realistic settings.

Funai and colleagues conducted a systematic review in the early 2000s to assess the distribution of study designs in four major journals of gynaecology and obstetrics [1]. They found that about 70% of the literature published has been based on observational study designs. This finding has been replicated by researchers in other disciplines who have that more publications reported the findings of observational studies than randomized studies [2] . The need for evidence synthesis is consequently not limited to reviews of randomized controlled trials. Moher and colleagues conducted a systematic review of 300 published systematic reviews and meta-analyses indexed in Medline in November 2004. They found, that 15% of all published systematic reviews and meta-analyses were based on observational research addressing questions such as aetiology, prognosis or diagnosis [3].

Importantly evidence synthesis of observational studies differ in many ways from conventional meta-analytical approaches of RCTs. The process from defining the research questions, conducting an adequate literature search and systematically review and synthesize evidence is more iterative in the context of systematic reviews of observational studies. Additionally, observational research is more prone to confounding and bias than randomized controlled studies which may modify the approach to evidence synthesis based on observational studies [4, 5]. The quality of systematic reviews and meta-analyses of observational research may be questionable. One potential reason for this is that guidelines on conducting evidence synthesis of observational studies are not well established. Stroup et al published in 2000 a guideline on how to report a systematic review or meta-analysis of observational studies without emphasising that conduct and reporting of research are two distinct concepts [6].

The COSMOS initiative was launched in order to improve the quality of systematic reviews and meta-analyses of observational studies. The presented study is a preparatory sub-study with the purpose of systematically reviewing existing documents dealing with methodological key issues likely to be encountered in the review process of observational research.

Objectives

The objective of this study is to review existing documents providing recommendations on how to synthesise evidence from observational studies. Specifically, we will explore:

1. what methodological key issues are addressed by documents
2. the extent of the explanation of the key issues covered in documents

Definition of guideline

The term guideline is widely used in the context of different professional fields and therefore the spectrum of its definition leads from vague expressed notions to highly specific conceptual determinations. The Oxford English Dictionary defines a guideline as a general rule, principle, or piece of advice (http://oxforddictionaries.com). The Collins English Dictionary describes the term as a principle put forward to set standards or determine a course of action (http://www.collinsdictionary.com) and the Encyclopaedia Britannica specifies it as an indication or outline of policy or conduct (http://www.britannica.com). In the medical context the term generally refers to clinical practice guidelines in which recommendations on the appropriate treatment and care of people with specific diseases and conditions (http://www.sign.ac.uk/about/introduction.html, http://www.iom.edu/, http://www.agreetrust.org/ http://guidance.nice.org.uk/CG,). Most of the definitions have in common that they suggest a guideline should aim at determining a course of action recommended for standard practice.

We adapted the above mentioned definitions and propose that a guideline on how to conduct a systematic review or meta-analysis of observational studies should:

1. define methodological key problems a reviewer might encounter on the review process (what are the problems?)
2. give step by step recommendations on how to address these methodological problems (what are the recommendations to solve the problems?)
3. explain the rational of these recommendations and provide methodological knowledge to put them into context (how should these recommendations be used and why?)
4. provide extensive references to be consulted for special methodological issues (what can you do if the recommendation doesn't answer your problem?)

Key problems to be addressed by a guideline on conducting evidence synthesis of observational studies

We will examine if and how documents identified by our search suggest address the following methodological key issues when performing evidence synthesis of observational studies (*see amendment 3, below*):

- Protocol
- Research question
- Search strategy
- Study eligibility
- Different study designs
- Data extraction
- Risk of bias assessment
- Between-study heterogeneity
- Publication bias
- Statistical analysis

Methods

Search strategy

In a first step we will search in Medline for relevant documents that provide recommendations on how to conduct a systematic review and meta-analysis of observational studies using a combination of the terms “systematic review”, meta-analysis, observational. In a second step we will perform web-based searches on specific websites of review centres including:

- the Cochrane Library (http://www.cochrane.org/)
- the Center for Reviews and Dissemination (http://www.york.ac.uk/inst/crd/index.htm)
- the Campbell Collaboration (http://www.campbellcollaboration.org/)
- the Scottish Intercollegiate Guidelines Network (http://www.sign.ac.uk/index.html)
- the Agency for Healthcare Research and Quality (http://www.ahrq.gov/)
- the EQUATOR Network (http://www.equator-network.org/)
- the National Institute for Health and Care Excellence (NICE) (http://www.nice.org.uk/)
- the Effective Public Health Practice Project (http://www.ephpp.ca/)

We will then screen all online issues of the following journals specifically focusing on evidence synthesis:

- Research Synthesis Methods (http://onlinelibrary.wiley.com/journal/10.1002/(ISSN)1759-2887/issues);

- Systematic Reviews (http://www.systematicreviewsjournal.com/archive);
- Epidemiologic Reviews (http://epirev.oxfordjournals.org/content/by/year).

During the study selection process additionally relevant articles will be identified through reference screening.

Eligibility

We will include all documents giving detailed methodological advice addressing at least one key issue about systematic reviews and meta-analyses of observational studies irrespective if they are considered as a guideline by the authors of the document or the reviewers involved in this systematic review. We will exclude articles if they (*see amendment 1, below*):

1. are itself systematic reviews or meta-analyses of observational studies, without reference to methodological aspects of systematic reviews and meta-analyses;
2. systematically assess methodological characteristics of already published systematic reviews or meta-analyses of observational studies, without reference to methodological aspects of systematic reviews and meta-analyses (e.g. reporting of search strategies in systematic reviews and meta-analyses of observational studies);
3. explicitly refer their methodological guidance to the conduct of systematic reviews or meta-analysis of *RCTs*
4. give methodological advice on how to *report* systematic reviews or meta-analyses of observational study
5. are text-book like repetitions of general principles not genuinely established by the authors, or are themselves books.

After a first selection of relevant literature based on abstract screening, two reviewers will check the remaining full-text for their eligibility. Disagreements, especially those concerning the decision to exclude a document will be resolved by discussion with a third senior methodologist.

Data extraction

Pairs of reviewers will be randomly assigned papers for data extraction. Text passages giving methodological advice on any of the above mentioned key issues will be extracted by both reviewers and passages agreed on by both reviewers will be entered into a consensus data extraction sheet. Disagreements will be resolved by discussion with a senior methodologist.

We will also explore to what extent these key issues are addressed (*see amendment 2, below*). This process is prone to subjectivity and therefore suggest the following grading system to be applied to classify how detailed the key issues are addressed:

- 0 not addressed: key issue is not mentioned;
- 1 briefly addressed: key issue is mentioned but no recommendation on what reviewers should do is given;
- 2 moderately addressed: explicit recommendation on the conduct of systematic reviews and meta-analyses of observational studies is given but recommendation might not be clear and comprehensive or based on clear reasoning;
- 3: fully addressed: clear and comprehensive recommendation with extensive discussion of the key issue is given

Summarizing the results

We will descriptively summarise findings and will not perform meta-analysis.

References

1. Funai, E.F., et al., *Distribution of study designs in four major US journals of obstetrics and gynecology.* Gynecol Obstet Invest, 2001. **51**(1): p. 8-11.
2. Scales, C.D., Jr., et al., *Clinical research and statistical methods in the urology literature.* J Urol, 2005. **174**(4 Pt 1): p. 1374-9.
3. Moher, D., et al., *Epidemiology and reporting characteristics of systematic reviews.* PLoS Med, 2007. **4**(3): p. e78.
4. Zwahlen, M., A. Renehan, and M. Egger, *Meta-analysis in medical research: potentials and limitations.* Urol Oncol, 2008. **26**(3): p. 320-9.
5. Egger, M., M. Schneider, and G. Davey Smith, *Spurious precision? Meta-analysis of observational studies.* BMJ, 1998. **316**(7125): p. 140-4.
6. Stroup, D.F., et al., *Meta-analysis of observational studies in epidemiology: a proposal for reporting. Meta-analysis Of Observational Studies in Epidemiology (MOOSE) group.* JAMA, 2000. **283**(15): p. 2008-12.
7. Higgins, J. and S. Green, *Cochrane Handbook. Version 5.1.0.* http://handbook.cochrane.org/, 2011.
8. Liberati, A., et al., *The PRISMA statement for reporting systematic reviews and meta-analyses of studies that evaluate healthcare interventions: explanation and elaboration.* BMJ, 2009. **339**: p. b2700.

Amendments

Number	Date	Change
1	March 2014	Decision to exclude studies published prior to 1994 because the development of systematic review methods began around then with the first publication of the Cochrane handbook.
2	August 2014	Decision not to classify the “extent” of recommendations or to analyse these data. This decision was made due to the subjective nature of these classifications and poor reviewer agreement on grades assigned (even with the explicit grading system outlined in the protocol). Documents were then classified only as “providing recommendations” or “not providing recommendations”.
3	September 2016	Decision to include documents that give guidance on how to report systematic reviews and meta-analyses of observational data. This decision was made because papers that gave guidance on reporting often give advice on one or more of the other key items.
