# Supplementary material for: Methods to systematically review and meta-analyse observational studies: a systematic scoping review of recommendations
Source: BMC Med Res Methodol. 2018 May 21;18:44. doi: 10.1186/s12874-018-0495-9 (PMC5963098; doi:10.1186/s12874-018-0495-9)
Supplement: Supplementary file 2 — Medline search terms. (PDF 100 kb) [file 12874_2018_495_MOESM2_ESM.pdf]

((epidemiological methods [MeSH Terms] OR guideline* OR recommend* OR comment [Publication Type] OR critical* apprais* OR standards))

AND

((“Meta-Analysis [Publication Type] OR “Meta-Analysis as Topic” [MeSH] OR meta-analysis OR metaanalysis OR “meta-analysis” OR “metaanalysis” OR “systematic review”)

AND

(observational OR non-randomized stud* OR case-control OR “case control” OR “case-control” OR cohort OR cross section* OR “cross sectional” OR “cross-sectional”))
