# Supplementary material for: Methods to systematically review and meta-analyse observational studies: a systematic scoping review of recommendations
Source: BMC Med Res Methodol. 2018 May 21;18:44. doi: 10.1186/s12874-018-0495-9 (PMC5963098; doi:10.1186/s12874-018-0495-9)
Supplement: Supplementary file 3 — Table S1. Sources of recommendations. (PDF 123 kb) [file 12874_2018_495_MOESM3_ESM.pdf]

Table S1: Sources of recommendations

Authors, year	Origin	Journal type	Affiliation of authors[1]
Abrams, 1995 [103]	CMR	Health sciences journal	Statistics or epidemiology department
Armstrong, 2007 [39]	Experts	Systematic review methods journal	Systematic review methods organisation
Ashford, 2009 [36]	Medline	Health sciences journal	Health sciences department
Austin, 1997 [76]	CMR	Epidemiology, statistics or general methods journal	Statistics or epidemiology department
Balshem, 2011 [85]	Medline	Epidemiology, statistics or general methods journal	Statistics or epidemiology department
Blair, 1995 [34]	Experts	Specialist medical journal	Clinical department
Brockwell, 2001 [117]	CMR	Epidemiology, statistics or general methods journal	Statistics or epidemiology department
Chaiyakunapruk, 2014 [54]	Medline	General medical journal	Health sciences department
Chambers, 2009 [32]	CMR	Epidemiology, statistics or general methods journal	Systematic review methods organisation
Colditz, 1995 [78]	CMR	Epidemiology, statistics or general methods journal	Statistics or epidemiology department
Davey Smith, 1997 [99]	Experts	General medical journal	Statistics or epidemiology department
Davey Smith, 1998 [97]	CMR	General medical journal	Statistics or epidemiology department
Doria, 2005 [104]	Medline	Specialist medical journal	Clinical department
Dwyer, 2001 [102]	CMR	Epidemiology, statistics or general methods journal	Statistics or epidemiology department
Egger, 1997a [29]	Experts	General medical journal	Statistics or epidemiology department
Egger, 1997b [98]	Experts	General medical journal	Statistics or epidemiology department
Fraser, 2006 [58]	CMR	Epidemiology, statistics or general methods journal	Statistics or epidemiology department
Friedenreich, 1994 [31]	CMR	Epidemiology, statistics or general methods journal	Statistics or epidemiology department
Furlan, 2006 [59]	CMR	Epidemiology, statistics or general methods journal	Statistics or epidemiology department
Golder, 2008 [60]	CMR	Epidemiology, statistics or general methods journal	Systematic review methods organisation
Greenland, 1994 [86]	CMR	Epidemiology, statistics or general methods journal	Statistics or epidemiology department
Guyatt, 2011a [96]	Medline	Epidemiology, statistics or general methods journal	Statistics or epidemiology department
Guyatt, 2011b [45]	Medline	Epidemiology, statistics or general methods journal	Statistics or epidemiology department
Guyatt, 2011c [94]	Medline	Epidemiology, statistics or general methods journal	Statistics or epidemiology department
Guyatt, 2011d [107]	Medline	Epidemiology, statistics or general methods journal	Statistics or epidemiology department
Hartemink, 2006 [110]	Medline	Epidemiology, statistics or general methods journal	Statistics or epidemiology department
Haynes, 2005 [57]	CMR	Epidemiology, statistics or general methods journal	Statistics or epidemiology department
Herbison, 2006 [93]	Medline	Epidemiology, statistics or general methods journal	Statistics or epidemiology department

Authors, year	Origin	Journal type	Affiliation of authors[1]
Hernandez, 2016 [108]	Medline	Health sciences journal	Statistics or epidemiology department
Higgins, 2013 [65]	Experts	Systematic review methods journal	Statistics or epidemiology department
Horton, 2010 [74]	Medline	Epidemiology, statistics or general methods journal	Statistics or epidemiology department
Ioannidis, 2011[89]	Medline	Epidemiology, statistics or general methods journal	Statistics or epidemiology department
Khoshdel, 2006 [30]	Medline	Specialist medical journal	Clinical department
Kuper, 2006 [62]	CMR	Epidemiology, statistics or general methods journal	Statistics or epidemiology department
Lau, 1997 [16]	Medline	General medical journal	Clinical department
Lemeshow, 2005 [63]	CMR	Epidemiology, statistics or general methods journal	Statistics or epidemiology department
Loke, 2011 [64]	Experts	Specialist medical journal	Statistics or epidemiology department
Loke, 2007 [35]	Medline	Epidemiology, statistics or general methods journal	Systematic review methods organisation
MacDonald-Jankowski, 2001 [46]	Medline	Specialist medical journal	Clinical department
Mahid, 2006 [55]	Medline	Specialist medical journal	Clinical department
Manchikanti, 2009 [47]	Medline	Specialist medical journal	Clinical department
Martin, 2000 [80]	CMR	Epidemiology, statistics or general methods journal	Statistics or epidemiology department
McCarron, 2010 [115]	Medline	Epidemiology, statistics or general methods journal	Statistics or epidemiology department
Moola, 2015 [41]	Medline	Epidemiology, statistics or general methods journal	Systematic review methods organisation
Moreno, 1996 [81]	CMR	Epidemiology, statistics or general methods journal	Statistics or epidemiology department
Munn, 2015 [72]	Medline	Epidemiology, statistics or general methods journal	Systematic review methods organisation
Naumann, 2007 [67]	Experts	Systematic review methods journal	Systematic review methods organisation
Normand, 1999 [48]	CMR	Epidemiology, statistics or general methods journal	Statistics or epidemiology department
Norris, 2013 [71]	Experts	Systematic review methods journal	Statistics or epidemiology department
O’Connor, 2014 [42]	Medline	Health sciences journal	Health sciences department
Pladevall-Vila, 1996 [101]	Medline	Epidemiology, statistics or general methods journal	Clinical department
Prevost, 2000 [118]	Medline	Epidemiology, statistics or general methods journal	Statistics or epidemiology department
Price, 2004 [49]	Medline	Specialist medical journal	Systematic review methods organisation
Raman, 2012 [50]	Medline	Epidemiology, statistics or general methods journal	Statistics or epidemiology department
Ravani, 2015 [43]	Medline	Specialist medical journal	Statistics or epidemiology department
Robertson, 2014 [95]	Journal	Systematic review methods journal	Health sciences department
Rosenthal, 2001 [51]	CMR	Specialist medical journal	Clinical department
Sagoo, 2009 [33]	CRD	General medical journal	Statistics or epidemiology department

Authors, year	Origin	Journal type	Affiliation of authors[1]
Salanti, 2005 [88]	Medline	Specialist medical journal	Statistics or epidemiology department
Salanti, 2009 [111]	Medline	Epidemiology, statistics or general methods journal	Statistics or epidemiology department
Sanderson, 2007 [91]	Experts	Epidemiology, statistics or general methods journal	Statistics or epidemiology department
Schünemann, 2013 [40]	Experts	Systematic review methods journal	Statistics or epidemiology department
Shamliyan, 2012 [90]	Experts	Epidemiology, statistics or general methods journal	Statistics or epidemiology department
Shuster, 2007 [119]	Medline	Epidemiology, statistics or general methods journal	Statistics or epidemiology department
Simunovic, 2009 [38]	Medline	Specialist medical journal	Clinical department
Smith, 1995 [112]	CMR	Epidemiology, statistics or general methods journal	Statistics or epidemiology department
Souverein, 2012 [82]	Medline	Epidemiology, statistics or general methods journal	Health sciences department
Stansfield, 2016 [68]	Journal	Systematic review methods journal	Health sciences department
Sterne, 2016 [83]	Experts	General medical journal	Statistics or epidemiology department
Stroup, 2000 [12]	CMR	General medical journal	Statistics or epidemiology department
Sutton, 2002a [79]	Medline	Specialist medical journal	Statistics or epidemiology department
Sutton, 2002b [100]	Medline	Epidemiology, statistics or general methods journal	Statistics or epidemiology department
Tak, 2010 [52]	Medline	Specialist medical journal	Clinical department
Takkouche, 1999 [105]	Medline	Epidemiology, statistics or general methods journal	Statistics or epidemiology department
Thomas, 2004 [53]	EPHPP	Health sciences journal	Health sciences department
Thompson, 2002 [116]	Medline	Epidemiology, statistics or general methods journal	Statistics or epidemiology department
Thompson, 2011 [113]	Medline	Epidemiology, statistics or general methods journal	Statistics or epidemiology department
Thompson, 2014 [69]	Journal	Systematic review methods journal	Health sciences department
Thornton, 2000 [61]	CMR	Epidemiology, statistics or general methods journal	Statistics or epidemiology department
Tufanaru, 2015 [44]	Medline	Epidemiology, statistics or general methods journal	Systematic review methods organisation
Tweedie, 1995 [114]	CMR	Epidemiology, statistics or general methods journal	Statistics or epidemiology department
Valentine, 2013 [75]	Experts	Systematic review methods journal	Statistics or epidemiology department
Verde, 2015 [84]	Journal	Systematic review methods journal	Statistics or epidemiology department
Weeks, 2007 [109]	CMR	Health sciences journal	Health sciences department
Wells, 2013 [37]	Experts	Systematic review methods journal	Statistics or epidemiology department
West, 2002 [92]	Experts	Health sciences journal	Statistics or epidemiology department
Wille-Jorgensen, 2008 [56]	Medline	Specialist medical journal	Clinical department
Winegardner, 2007 [66]	Medline	Specialist medical journal	Health sciences department

Authors, year	Origin	Journal type	Affiliation of authors[1]
Wong, 2008 [87]	Medline	Epidemiology, statistics or general methods journal	
Wong, 1996 [70]	CMR	Health sciences journal	Health sciences department
Zeegers, 2000 [106]	Medline	General medical journal	not specified
Zingg, 2016 [73]	Medline	Epidemiology, statistics or general methods journal	Health sciences department
Zwahlen, 2008 [77]	Medline	Specialist medical journal	Statistics or epidemiology department

1 Affiliation assessment based on first and last author affiliations. If affiliations fit in to 2 or more categories,“ systematic review methods organisation“ is given priority over other categories, then “statistics or epidemiology department“, then “clinical department“, then “other health sciences department”.

CMR: Cochrane Methodology Register
